# Amniotic Membrane Transplantation Preserves Vision in Pediatric Recessive Dystrophic Epidermolysis Bullosa: Case Series

**DOI:** 10.3390/jcm15093503

**Published:** 2026-05-03

**Authors:** Seika Den, Yukako Abukawa, Nanami Kishimoto, Ryuichi Shimada, Yuka Higashi, Kozue Kasai, Tadashi Nakano

**Affiliations:** 1 The Department of Ophthalmology, The Jikei University Katsushika Medical Center, 6-41-2, Aoto, Katsushika City 1258506, Tokyo, Japan; 2 The Department of Anesthesiology, The Jikei University School of Medicine, 3-19-18, Nishishinbashi, Minato City 1050003, Tokyo, Japan; yukakoabukawasandy@yahoo.co.jp; 3 The Department of Ophthalmology, The Jikei University School of Medicine, 3-19-18, Nishishinbashi, Minato City 1050003, Tokyo, Japanshimady.ryu@gmail.com (R.S.);

**Keywords:** recessive dystrophic epidermolysis bullosa, ocular surface reconstruction, amniotic membrane transplantation, amblyopia, general anesthesia, case series

## Abstract

**Background**: Recessive dystrophic epidermolysis bullosa (RDEB) is a rare inherited disorder characterized by extreme epithelial fragility and progressive cicatrization, frequently leading to severe ocular surface disease and early visual impairment. Surgical interventions such as ocular surface reconstruction (OSR) in childhood are often delayed because of anesthetic risks and concerns regarding recurrence. Consequently, the effectiveness of OSR, including amniotic membrane transplantation (AMT), and its impact on visual development remain poorly documented. **Methods**: We report a case series of two pediatric patients (three eyes) with genetically confirmed RDEB who underwent single-step OSR using AMT. Clinical outcomes, long-term visual acuity, perioperative management, and histopathological findings were evaluated. **Results**: Ocular manifestations included corneal epithelial damage, symblepharon, and pseudopterygium extending over the cornea. One patient underwent symblepharon lysis, superficial keratectomy, and AMT onto the bare sclera in the right eye at age 4 and in the left eye at age 8, both under intubated general anesthesia. The other patient underwent the same procedure in the right eye at age 6. Best spectacle-corrected visual acuity improved from ≤20/300 to 20/30 in all eyes, and pupillary zone clarity was maintained during the follow-up period (up to 6 years). Histopathology confirmed pseudopterygium with squamous metaplasia, goblet cell loss, and fibrovascular stroma. Safe general anesthesia was achieved through meticulous multidisciplinary perioperative planning involving anesthesiologists, dermatologists, and pediatricians. No systemic complications related to anesthesia or perioperative management were observed. **Conclusions**: Single-step OSR with on-lay AMT can restore and preserve visual function in pediatric RDEB. Early surgical intervention may prevent profound amblyopia and provide durable ocular surface stability. A multidisciplinary approach enables safe general anesthesia and perioperative management.

## 1. Introduction

Epidermolysis bullosa (EB) is a heterogeneous group of inherited mechanobullous disorders caused by mutations in the structural proteins of the dermal–epidermal junction [1,2,3,4]. Among its subtypes, recessive dystrophic EB (RDEB) is one of the most severe forms, resulting from biallelic mutations in the COL7A1 gene, which encodes type VII collagen, the main component of anchoring fibrils. Patients with RDEB develop generalized blistering and scarring of the skin and mucous membranes from birth, often accompanied by multisystem complications including growth retardation, anemia, esophageal strictures, skeletal deformities, congestive heart failure, and cardiomyopathy [5,6,7]. The lesions also affect the oral cavity, showing oral blisters, tooth destruction, and microstomia [8,9].

Repeated epithelial blistering caused by a lack of adherence and disruption of the corneal or conjunctival epithelia can lead to epithelial damage, limbal stem cell deficiency (LSCD), pseudopterygium, symblepharon, ectropion, and corneal scarring [10,11,12,13]. These ocular complications can cause profound visual impairment from early childhood. These cicatricial changes often progress, interfering with visual development and the quality of life. A major barrier to early surgical intervention in pediatric RDEB is the challenge of general anesthesia [14,15,16,17,18]. Consequently, many patients are deprived of timely ocular surgery despite progressive visual deterioration.

Amniotic membrane transplantation (AMT) has been an established technique for ocular surface reconstruction (OSR) in cicatrizing conjunctival and corneal disorders, including recurrent pterygium, symblepharon, persistent epithelial defects, and limbal stem cell deficiency, since the 1990s because of its anti-inflammatory, anti-fibrotic, and epithelialization-promoting properties [19,20,21,22,23,24,25]. In Japan, AMT has been covered by national health insurance since 2014. The membrane is obtained from donors undergoing elective cesarean delivery, processed and cryopreserved under sterile conditions, and distributed through certified Amniotic Membrane Banks. Very few reports have noted that AMT can help reconstruct the damaged conjunctival and corneal surface and prevent the recurrence of symblepharon in patients with EB [26,27,28,29].

Here, we report a case series of two pediatric patients with RDEB who underwent successful OSR with AMT. We evaluated the surgical outcomes and highlighted the role of early intervention in restoring vision in high-risk cases.

## 2. Methods

Three eyes of two pediatric patients with genetically confirmed RDEB underwent OSR, including symblepharon lysis, superficial keratectomy, and AMT. The clinical characteristics, surgical details, and long-term visual outcomes were reviewed retrospectively. Histopathological analysis was also performed. Written informed consent for publication of clinical information, surgical images, and photographs was obtained from the parents of all patients. They were informed that the article would be published as open-access content freely available to the public worldwide. This study adhered to the tenets of the Declaration of Helsinki. Our institution does not require ethical approval for reporting individual cases or case series.

### 2.1. Ophthalmological Examinations

Slit-lamp biomicroscopy with fluorescein staining was performed at each visit. The ocular media and fundus were examined under mydriasis; in eyes where corneal opacity precluded adequate visualization, these assessments were performed intraoperatively. AS-OCT (CASIA2, Tomey Corporation, Nagoya, Japan) was also performed. BSVA was measured using Landolt ring optotypes. Intraocular pressure was measured using a rebound tonometer (Icare IC200; M.E. Technica, Tokyo, Japan), which requires neither topical anesthesia nor forced eyelid opening and is therefore suitable for patients with RDEB who have fragile eyelids or limited palpebral aperture, unlike conventional applanation or non-contact tonometry.

### 2.2. Surgical Procedure

Before surgery, an anesthesiologist and each patient’s attending dentist conducted a medical evaluation to assess the feasibility of general anesthesia with endotracheal intubation. A detailed simulation was performed to confirm perioperative management, including intravenous access and fluid administration, in consultation with the pediatricians and dermatologists. General anesthesia was administered by a highly skilled anesthesiologist to all patients.

An incision was made along the lid margin to release the symblepharon and free the globe, after which the eye speculum was set. The fibrous tissues covering the cornea were removed and served for pathological analysis. Then the scarred conjunctiva and subconjunctival fibrous tissues were removed as extensively as possible, exposing the bare sclera over approximately the temporal half of the ocular surface. Finally, an amniotic membrane was transplanted onto the bare sclera in an on-lay fashion. The amniotic membrane was placed with the stromal side facing down toward the scleral surface and the epithelial (basement membrane) side facing upward and secured with interrupted 8-0 polyglactin 910 absorbable sutures to cover the entire area of the excised conjunctiva. A bandage contact lens was placed on the cornea at the end of the surgery. Punctal plugs were inserted into both the upper and lower puncta at the end of the surgery for the patient in Case 1.

At the end of the surgery, a smooth corneal surface and free fornices were confirmed. Ophthalmological assessment during surgery revealed a normal lens, intact optic disc, and fundus in all eyes. Administration of levofloxacin 1.5% and preservative-free betamethasone 0.1% was initiated four times a day after the surgery. After complete epithelialization, these eye drops were switched to fluorometholone 0.1% and tranilast 0.5%.

We did not perform temporary amniotic membrane patching over the cornea because postoperative removal of the amniotic membrane under topical anesthesia was predicted to be difficult due to their age and condition.

**Figure 1:**
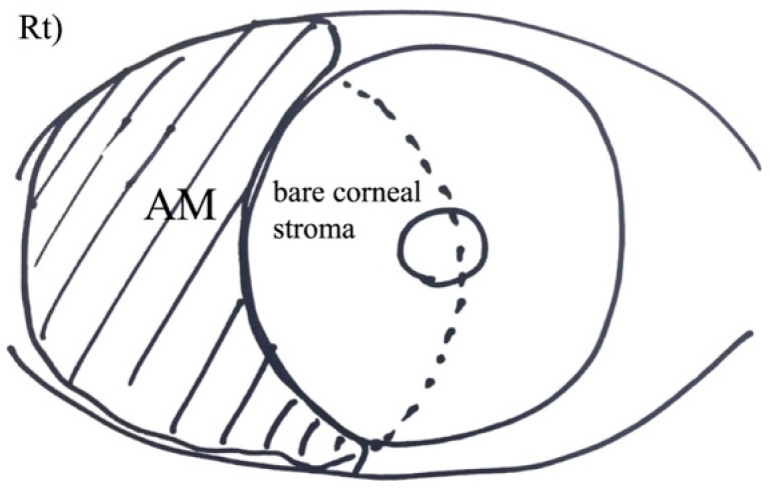
Schematic diagram of the on-lay amniotic membrane transplantation (AMT) technique. Following symblepharon lysis, superficial keratectomy, and subconjunctival fibrous tissue removal, the amniotic membrane is placed onto the bare sclera with the stromal side down and the basement membrane side up and secured with interrupted absorbable sutures.

A schematic diagram illustrating the on-lay AMT technique is provided in Figure 1.

### 2.3. Histopathological Analysis

Excised pseudopterygium tissue obtained during surgery in Case 1 was fixed in 10% neutral buffered formalin, processed through graded alcohols, and embedded in paraffin. Sections of 4 µm thickness were cut and stained with hematoxylin and eosin (H&E) according to standard protocols. Slides were examined by light microscopy to assess epithelial morphology, goblet cell density, and stromal characteristics.

## 3. Results

### 3.1. Case 1

A Japanese boy was referred to us at the age of 3 years by his pediatrician for corneal opacification that had developed since the age of 2. He was born with generalized dermolytic blister formation and was immediately diagnosed with RDEB by skin biopsy and genetic testing, which identified a COL7A1 mutation. Thereafter, he was followed up by a dermatologist, pediatrician, and dentist.

Physical examination revealed pseudosyndactyly and claw-like contracture of both hands and feet. He had difficulty ambulating. Active and chronic blisters, erythematous lesions, and scarring were noted on his entire body. Laboratory investigations revealed chronic iron-deficiency anemia requiring oral iron replacement and periodic blood transfusions based on hemoglobin level, and nutritional supplementation with an oral enteral formula was ongoing. No systemic corticosteroids or immunosuppressive agents were in use, and the patient had been in a clinically stable condition for several years prior to surgery. The patient also had oral manifestations.

**Figure 2:**
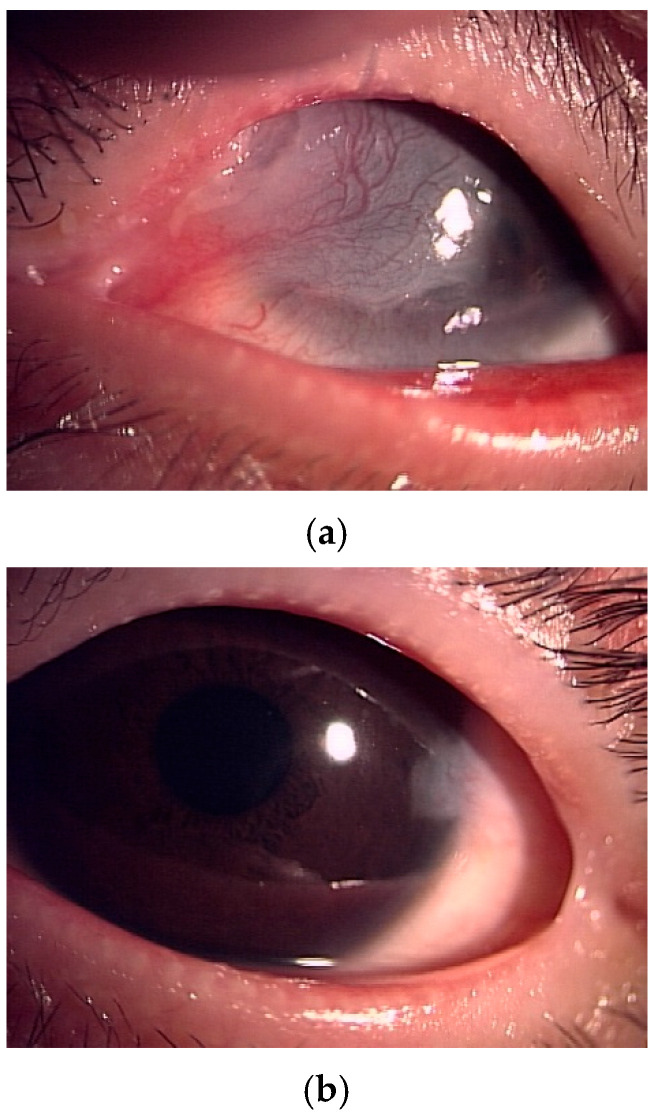
(**a**) Case 1, right eye at the initial visit: supratemporal symblepharon with associated pseudopterygium with pannus extending onto the cornea and causing limited ocular motility and abnormal blinking. (**b**) Case 1, left eye at the initial visit: slight temporal pseudoterygium with limited corneal opacity.

**Figure 3:**
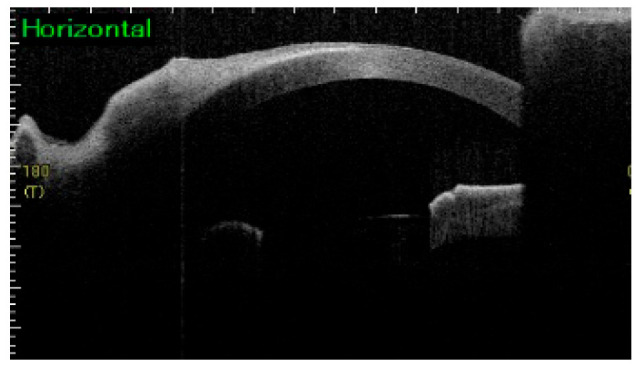
Anterior segment optical coherence tomography of the right eye in Case 1 before surgery, showing a pseudopterygium covering half of the cornea but without stromal involvement.

In the right eye, a temporal–upper symblepharon was observed, resulting in limited ocular motility and abnormal blinking (Figure 2a). Pseudopterygium extending over half of the cornea was observed, while anterior segment optical coherence tomography (AS-OCT) revealed no stromal involvement (Figure 3). The best spectacle-corrected visual acuity (BSVA) was 20/300. In the left eye, a slight temporal symblepharon was observed, and the BSVA was 20/30 (Figure 2b). Decreased tear secretion and corneal epithelial damage were observed in both eyes.

We performed OSR using AMT in the right eye at the age of 4 years under general anesthesia with an endotracheal tube. Conjunctival epithelialization onto the amniotic membrane and corneal epithelialization were noted within the first 2 weeks after the surgery. Fluorometholone and tranilast eye drops were administered postoperatively to prevent symblepharon progression. Lubricating eye drops (diquafosol sodium and hyaluronate) and antibiotic ointments were also used for ocular surface management. During follow-up, dendritic keratitis developed simultaneously in both eyes at the age of 7 years, which resolved within 1 week with acyclovir ointment.

**Figure 4:**
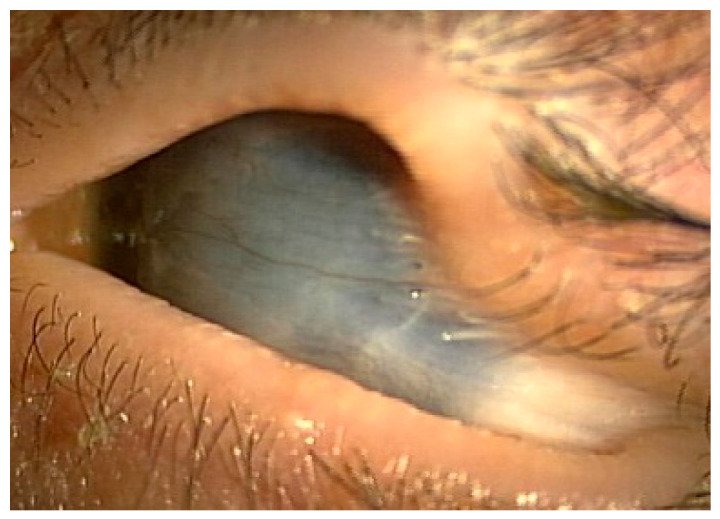
Case 1, left eye, before surgery: severe temporal symblepharon and pseudopterygium covering the entire cornea were noted.

As temporal symblepharon and pseudopterygium slowly progressed and BSVA dropped to hand motion in the left eye (Figure 4), the patient underwent the same surgery in the left eye at age 8.

**Figure 5:**
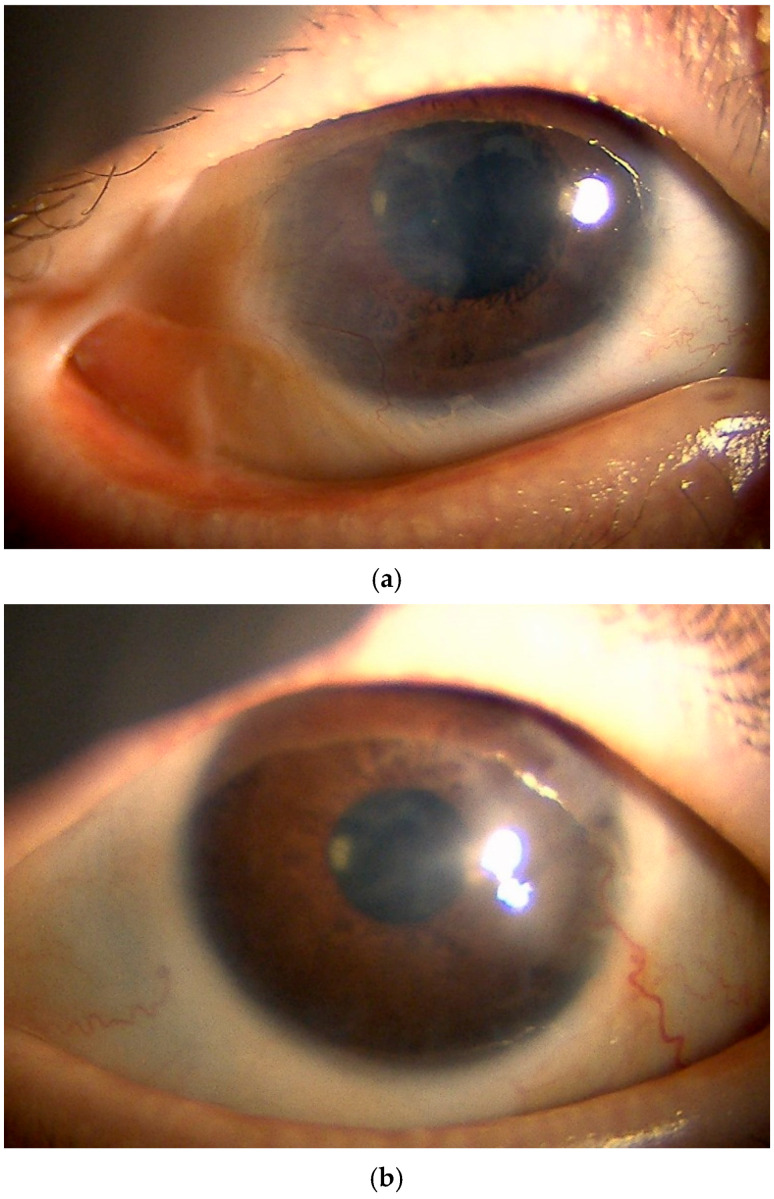
(**a**) Case 1, right eye after surgery: corneal clarity was preserved in the pupillary area, and a mild recurrence of temporal symblepharon and slight corneal opacity were noted without visual compromise 6 years postoperatively. (**b**) Case 1, left eye after surgery: corneal clarity was preserved in the pupillary area, whereas slight corneal opacity without visual compromise was observed 2 years postoperatively.

At the latest follow-up, 6 years postoperatively in the right eye and 2 years in the left, the BSVA was 20/30 in both eyes. The pupillary area remained clear, and ocular motility and blinking were intact despite a slight recurrence of the temporal symblepharon in the right eye (Figure 5a,b). No elevation of intraocular pressure was observed during the follow-up period. The patient attended a mainstream primary school and was able to engage in learning using a tablet device.

**Figure 6:**
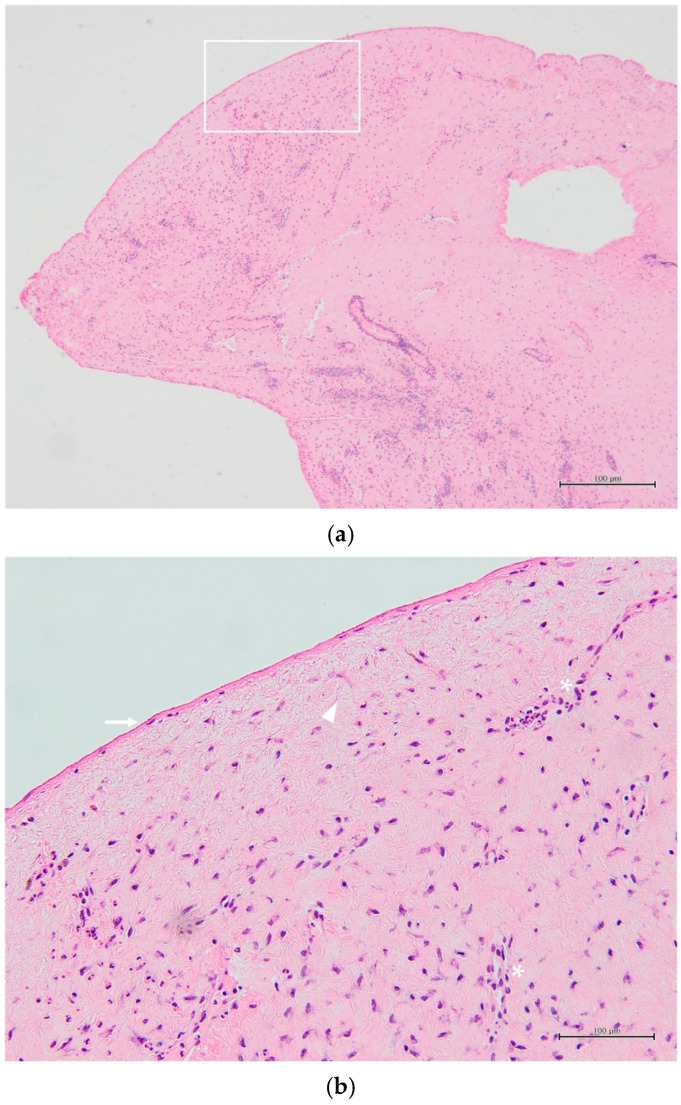
(**a**) Histopathological section of excised pseudopterygium tissue from Case 1, showing non-keratinizing stratified squamous epithelium with squamous metaplasia, absence of goblet cells, and underlying fibrovascular stroma with collagen deposition. (**b**) Higher magnification of the white boxed area in (**a**), showing epithelial changes including squamous metaplasia and absence of goblet cells (arrow), subepithelial collagen deposition (arrowhead), and fibrovascular proliferation (asterisks). Scale bar = 100 µm.

Histopathological examination revealed non-keratinizing stratified squamous epithelium consistent with squamous metaplasia, without goblet cells. The subepithelial stroma was composed of fibrous connective tissue with collagen deposition, mild capillary proliferation, and lymphocyte infiltration. These findings were consistent with those of a pseudopterygium (Figure 6a,b).

### 3.2. Case 2

A Japanese boy with genetically confirmed RDEB due to a COL7A1 nonsense mutation (exon 6, c.793C>T) diagnosed shortly after birth presented to our institution at the age of 6 for surgical management of ocular complications that had developed at 1 year of age. Prior to presentation, he had been treated with topical lubrication and 0.1% fluorometholone prescribed by his referring ophthalmologist. He had congenitally corrected transposition of the great arteries and pulmonary artery stenosis. Laboratory investigations revealed chronic iron-deficiency anemia and hypoalbuminemia, consistent with the systemic nutritional compromise characteristic of severe RDEB. Oral and pharyngeal blistering caused dysphagia, and enteral nutritional supplementation was ongoing. The pulmonary artery stenosis had decreased in severity over time and did not require intervention; cardiac function was managed with oral bisoprolol. No systemic corticosteroids or immunosuppressive agents were in use, and the patient had been in a clinically stable condition for several years prior to surgery. He was followed up by a pediatrician, cardiologist, dermatologist, and dentist.

Temporal symblepharon, pseudopterygium, and corneal opacification were observed in both eyes, with a more severe condition in the right eye. Tear volume was adequate, with no evidence of corneal epithelial damage. BSVA was 20/200 in both eyes. Similarly, he underwent OSR using AMT in the right eye at age 6, and BSVA improved to 20/30 at 8 months postoperatively. The patient continued using fluorometholone 0.02% and tranilast eye drops to prevent the proliferation of subconjunctival fibrous tissue. No elevation of intraocular pressure was observed during the follow-up period.

Although temporal pseudopterygium slightly recurred in the right eye 3 years after surgery, the pupil area remained clear and the BSVA remained at 20/30. The patient attended a mainstream primary school. Surgery on the left eye was planned, as temporal pseudopterygium and corneal opacity extending over half of the cornea had slowly progressed despite the continued use of steroid and tranilast eye drops.

The clinical courses of both cases are summarized in Table 1.

## 4. Discussion

Our series demonstrates that single-step OSR with AMT can be safely and effectively performed in pediatric patients with RDEB. Previous reports have described surgical management of ocular involvement in RDEB; however, the surgical concepts and the role assigned to the amniotic membrane differ substantially from those of the present study. Altan-Yaycioglu et al., Goyal et al., and Koulisis et al. reported favorable outcomes using the amniotic membrane as a temporary corneal patch combined with palpebral conjunctival suturing for fornix reconstruction [26,27,28]. However, these approaches frequently required additional mechanical measures, such as symblepharon rings, silicone sheets, or tarsorrhaphy, to prevent re-adhesion, and were applied in relatively older patients, including adolescents. It is therefore possible that the favorable outcomes observed in these studies were influenced by both adjunctive procedures and the more mature ocular surface environment. In contrast, our approach utilized the amniotic membrane transplanted onto the bare sclera as a bulbar conjunctival substrate, enabling single-step ocular surface reconstruction without palpebral conjunctival suturing or mechanical barriers. Specifically, the amniotic membrane was placed in an on-lay fashion, with the stromal side opposed to the bare sclera and the basement membrane side facing outward, serving as a scaffold for conjunctival epithelialization over the reconstructed bulbar surface. This approach differs fundamentally from in-lay techniques, in which the membrane is buried beneath existing tissue, and from sandwich configurations; rather, it functions as a permanent conjunctival graft that directly replaces the excised scarred tissue. These findings suggest that restoration of the bulbar conjunctival epithelium, rather than palpebral reconstruction alone, may play a key role in suppressing symblepharon recurrence. Thanos et al. reported successful ocular surface reconstruction using cultivated limbal epithelial transplantation on an amniotic membrane scaffold; however, this approach may limit its applicability in patients with bilateral involvement [29]. A comparison of published surgical approaches and outcomes is summarized in Table 2.

This technique can be regarded as an adaptation of methods previously used for recurrent pterygium, in which the amniotic membrane is placed on the scleral surface to suppress inflammation and fibrovascular proliferation while supporting conjunctival epithelialization [21,22]. In the context of RDEB, this concept is particularly rational, as the amniotic membrane contains type VII collagen and may partially compensate for the underlying molecular deficiency associated with epithelial fragility [30]. Unlike recurrent pterygium, however, ocular surface stability in RDEB cannot be achieved by surgery alone and requires meticulous long-term postoperative management. In our cases, topical fluorometholone and tranilast were administered to suppress chronic inflammation and fibrotic proliferation, together with aggressive lubrication using diquafosol sodium and hyaluronate eye drops. In addition, punctal occlusion was employed to optimize tear film stability. Although the individual contribution of each component cannot be determined in this small series, such comprehensive postoperative care may be essential for maintaining epithelial integrity and limiting recurrence in RDEB.

Pseudopterygium is a common ocular manifestation of RDEB. In our cases, this was corroborated not only by AS-OCT but also by histopathological analysis, which demonstrated non-keratinizing conjunctival epithelium with squamous metaplasia and fibrovascular stroma, consistent with the pseudopterygium.

In our patients, a clear pupillary axis and useful vision were maintained for up to 6 years postoperatively despite mild recurrence. Improvement in visual acuity during the critical developmental period likely contributed to the prevention of severe amblyopia and supported normal educational development. Although a 6-year follow-up may appear limited for a lifelong condition, this interval encompasses the critical period of visual development in childhood, during which untreated ocular surface disease carries the greatest risk of irreversible amblyopia. Maintaining pupillary axis clarity and functional vision throughout this period is therefore clinically meaningful, even though long-term recurrence remains possible and requires continued monitoring.

General anesthesia for pediatric patients with RDEB is a major barrier to surgical intervention owing to difficulties in vascular access due to skin fragility, airway management issues from mucosal and oral involvement, and increased risk of iatrogenic injury. Our experience highlights that meticulous preoperative planning, including multidisciplinary simulations with dermatologists, pediatricians, dentists, and anesthesiologists, can allow safe anesthetic administration and perioperative management. Specific measures included modifying all adhesive monitors to reduce skin contact, securing intravenous access at a preoperatively identified skin-intact site without adhesive tape, introducing a warmed, lubricated RAE tube via fiberoptic bronchoscopy to minimize mucosal trauma with the tube anchored to a circuit stand rather than taped to the face, and combining continuous dexmedetomidine infusion with deep extubation to prevent emergence agitation.

Preventing the recurrence of pterygium and symblepharon caused by progressive LSCD remains a major challenge. Mitomycin C (MMC) has been considered an adjuvant option; however, we did not apply MMC because of concerns regarding potential long-term complications, such as scleral melt.

For bilateral LSCD, cultivated oral mucosal epithelium transplantation (COMET) has been developed [31,32]. In Japan, autologous COMET using an amniotic membrane substrate has recently been approved for clinical use, which may represent a potential future option for RDEB-associated LSCD [33]. However, further studies are needed because RDEB has oral manifestations that require attention.

Recently, gene therapy has emerged as a potential therapeutic strategy for RDEB-associated ocular surface disease. Beremagene geperpavec (B-VEC), a replication-deficient herpes simplex virus type 1-based vector delivering a functional COL7A1 gene, has been developed to restore type VII collagen expression. In a recent clinical report, topical ophthalmic administration of B-VEC following surgical symblepharon lysis promoted corneal epithelial healing and resulted in marked visual improvement without significant ocular adverse events [34]. These findings suggest that gene-based approaches targeting the underlying molecular defect may represent a promising future adjunct for ocular surface reconstruction in RDEB, although evidence remains limited and further studies are required to determine long-term safety and efficacy.

Although the number of reported cases is very limited and the long-term prognosis remains uncertain, our findings suggest that carefully selected early surgical intervention can be a feasible therapeutic option for sight-threatening ocular surface complications in pediatric RDEB. If early intervention in childhood prevents profound amblyopia, subsequent recurrence of pseudopterygium may be managed with repeat surgery under local anesthesia once the patient has reached an age appropriate for such procedures.

This study is limited by the small number of cases and its retrospective design; however, it provides clinically meaningful insights into a rare and challenging condition.

## 5. Conclusions

In pediatric RDEB, symblepharon, pseudopterygium, and corneal opacity with pannus causing progressive visual impairment can be effectively managed with single-step ocular surface reconstruction using on-lay amniotic membrane transplantation, achieving successful restoration of the ocular surface and meaningful visual acuity gains. This procedure can be safely performed in pediatric patients with RDEB when supported by meticulous multidisciplinary perioperative management. Early surgical intervention not only preserves pupillary clarity and visual function but also plays a critical role in preventing amblyopia and supporting developmental outcomes. Although our series is limited in size, these findings highlight AMT as a valuable vision-preserving option for sight-threatening ocular complications of this rare and challenging condition.

## Figures and Tables

**Table 1:** Clinical course, surgical interventions, and outcomes for both cases.

	Age at Event	Clinical Findings	Surgical Intervention	BSVA	Complications
**Case 1** **Rt**	3 y	Temporal symblepharon and pseudopterygium, over half of the corneal opacity, limited motility		20/300	
	4 y		SL + SK + AMT		
	7 y			Bil herpetic keratitis	
	10 y	Slight temporal symblepharon, clear pupillary zone		20/30	
**Case 1** **Lt**	3 y	Slight pseudopterygium		20/30	
	8 y	Temporal symblepharon and pseudopterygium, entire corneal opacity, limited motility	SL + SK + AMT	HM	
	10 y	Slight temporal pseudopterygium, clear pupillary zone	20/30	none	
**Case 2** **Rt**	6 y	Temporal symblepharon and pseudopterygium, over half of the corneal opacity, limited motility	SL + SK + AMT	20/200 *	
	9 y	Slight pseudopterygium, clear pupillary zone	20/30	none	
**Case 2** **Lt**	6 y	Temporal pseudopterygium and corneal opacity		20/200	
	9 y	Temporal symblepharon and pseudopterygium, over half of the corneal opacity	Planned	20/200	

Rt, right; Lt, left; Bil, bilateral; BSVA, best spectacle-corrected visual acuity; SL, symblepharon lysis; SK, superficial keratectomy; AMT, amniotic membrane transplantation; HM, hand motion. *, BSVA measured at presentation, same year as surgery.

**Table 2:** Comparison of surgical approaches and outcomes of ocular surface reconstruction in RDEB.

Study	Age (y)	Patients /Eyes	Surgical Procedure	AM Role	Adjunctive Measures	Follow-Up
Altan-Yaycioglu et al., 2006 [26]	12	1/1	SL + SK + AMT	Temporal corneal patch, palpebral conjunctival suture	Symblepharon ring	22 months
Goyal et al., 2006 [27]	Mean 8.7 (4–16)	4/5	SL + SK + AMT	Palpebral conjunctival suture	Silicone sheet, temporary tarsorrhaphy	Mean 18.3 months (12–29)
Koulisis et al., 2016 [28]	17	1/2	SL + SK + AMT	Temporal corneal patch, palpebral conjunctival suture	None	14 years
Thanos et al., 2010 [29]	11.5	1/1	Auto CLET	Substrate for cultivated epithelia	Autologous serum drops, subconjunctival bevacizumab	28 months
Present study	4–8	2/3	SL + SK + AMT	On-lay graft onto bare sclera	None	Up to 6 years

SL: symblepharon lysis. SK: superficial keratectomy. AMT: amniotic membrane transplantation. CLET: cultivated limbal epithelial transplantation.

## Data Availability

The original contributions presented in this study are included in the article. Further inquiries can be directed to the corresponding author.
